# Trust in the European Union project and the role of ECB

**DOI:** 10.1007/s40888-021-00229-5

**Published:** 2021-04-20

**Authors:** Giorgio Liotti, Rosaria Rita Canale

**Affiliations:** 1 Department of Political Sciences, University of Naples “Federico II”, Via Rodinò 22, 80136 Naples, Italy; 2 Department of Business and Economics, University of Naples “Parthenope”, Via Generale Parisi, 13, 80132 Naples, Italy

**Keywords:** Trust, European Union, Monetary policy, Systemic shock, Dynamic panel data, E02, E58, E63, C33

## Abstract

Was the European Central Bank able to assure the relaunch of the European project after the weakening of the post-crisis period? To answer this question, this paper presents an empirical analysis connecting citizen trust in the European Union with a variable intended to be a measure of the monetary policy strategy of the European Central Bank, namely, the interest rate on government bonds extracted from the 1-year maturity yield curve. The dynamic panel technique, applied to nineteen Eurozone countries for the time span of 2004–2018, estimates the presence of a long-run common relationship between the variables despite allowing different short-run adjustment mechanisms. Results are revealed to be not univocal: the easy monetary policy strategy is associated for the whole period with a decline of trust, and therefore, despite its impressiveness, it was not sufficient to relaunch the European Union project. However, when considering the change in strategy of the post-2013 period, it seemed to have contributed to a slight inversion of the decline of trust. These results highlight the importance of non-conventional measures and call for further support from coordinated policy action as a response to the negative shock deriving from the COVID-19 pandemic.

## Introduction

The Eurozone has been experiencing, since the 2008 financial crisis, a particularly delicate phase of its history. The occurrence of the systemic shock coming from the crisis required unprecedented responses that involved both national fiscal policies and the centralized monetary policy. However, national fiscal policies had a different fiscal space that, in turn, reflected on divergent interest rates and different degrees of sustainability of public accounts. Given the policy structure of the Eurozone, the systemic shock produced asymmetrical effects that reflected on the hold of the European project. In the absence of a sense of common destiny (Baldwin and Wyplotz 2019), centrifugal forces, asking for greater autonomy in the management of economic policy, were set in motion, prefiguring the possibility of a dissolution of the monetary union. The situation appears to be very similar to what is happening in these times of the COVID-19 systemic shock and therefore are a good laboratory to investigate the strength of the European Union (EU) project.

**Fig. 1:**
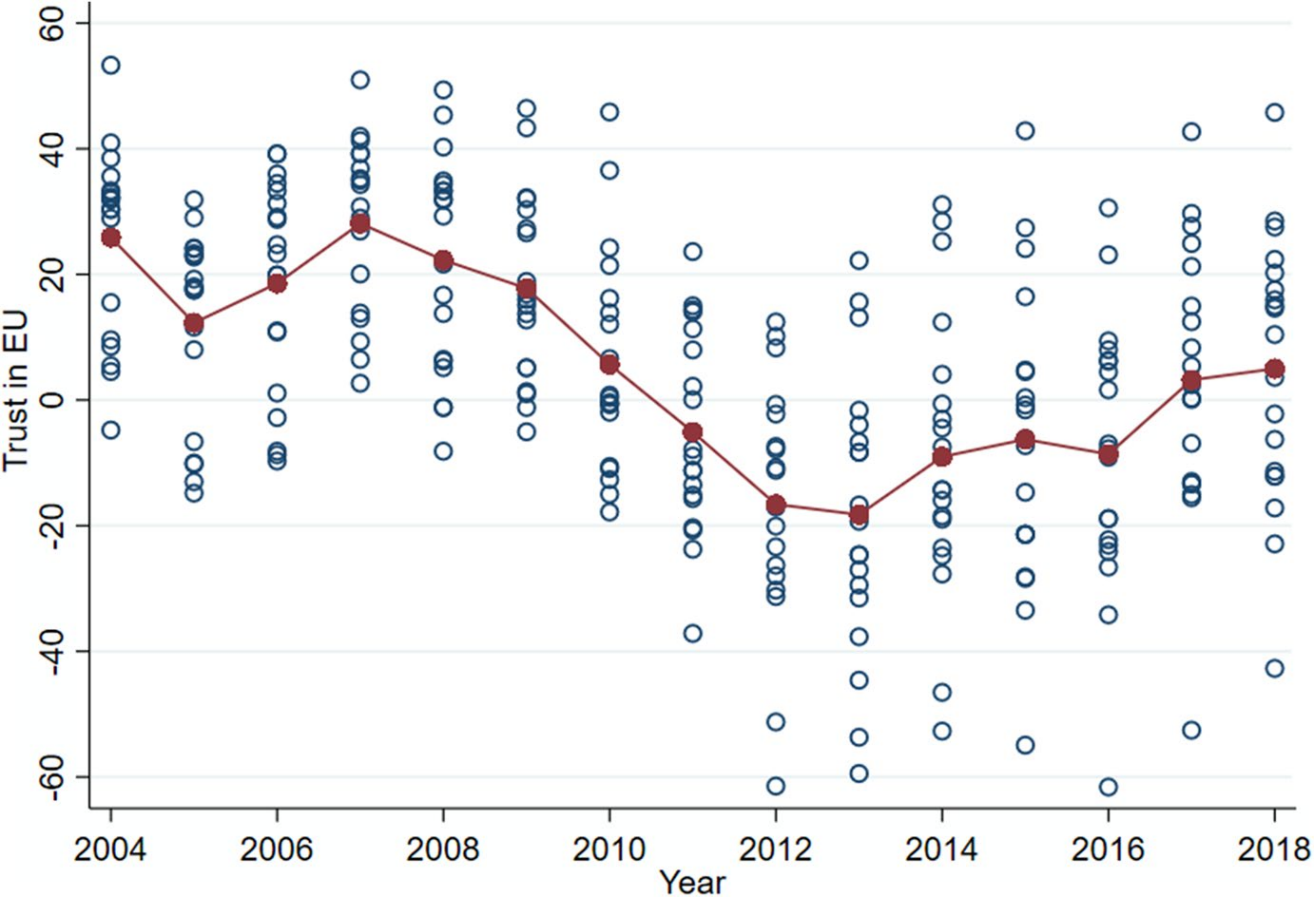
Net trust in EU from 2004–2018 in 19 Eurozone countries. *Source*: Authors’ calculation on Eurobarometer dataset

With the objective of creating an integrated and solid monetary union, the European Commission (EC) has been monitoring public opinion trends in its institutions within the Member States via the Eurobarometer.^1^ Essentially a biannual survey, the Eurobarometer covers a wide range of topics, including questions ascertaining the extent to which European citizens tend to trust in their main decision-making bodies, thus monitoring the process of integration and legitimacy. Here, net trust in the EU is considered as a variable capturing the degree of citizens’ confidence in the supranational project. Figure 1 presents the “Net trust” in EU countries, which is calculated as the difference between those who trust and those who do not trust in the EU from 2004 to 2018 for 19 Eurozone countries, both in its panel mean (continuous line) and in its value by country (circles). As Fig. 1 shows, this degree of confidence, after an initial enthusiasm lasting more or less until 2007 (+ 30% in average), seems to have declined in subsequent years until 2012 (− 20% in average).

At the end of 2012, the governor of the European Central Bank (ECB), Mario Draghi, declared that he would have saved the Euro “whatever it takes”—announcing a monetary policy strategy accounting for financial pressure and growth divergence—and in just a few words, he was able to definitely halt the threat of the dissolution of the monetary union. The very low interest rates and the massive injection of liquidity through open market operations increased national fiscal space and promised to reduce growth differences across countries. Starting from 2013, trust in the European Union seemed to increase once again, reaching the positive level of about 5% in the average. This inversion seems to signal a revised role of the ECB in supporting the institutional consolidation project.

Was the policy strategy of the Eurozone—centralized monetary policy associated with national fiscal discipline—able to assure the holding of the European project? Was the ECB intervention enough to save the Eurozone as a political entity after the weakening of the post-crisis period? To answer these questions, this paper connects citizens’ trust in the European Union with the monetary policy strategy with the aim to evaluate if its strategy was a cohesion instrument and if it was able to convince European citizens that the European project is something to rely on. Therefore, the main dependent variable is the interest rates extracted from the 1-year maturity yield curve on sovereign bonds. The choice of this variable relies on its ability to capture the monetary policy strategy as a whole, as it includes both the ECB interest rate setting policy and the effects of open market operations implemented from the assignment to Draghi the role of ECB governor.

The aim of the paper is therefore—rather than to search for the determinants of trust—to investigate the role of the ECB in granting trust in the European Union project and to convince European citizens that it is worthy of their trust. At the centre stage of the investigation is the trustworthiness of the political project rather than issues related to the common currency, which can be value-based rather than driven by a sense of belonging (Bergbauer et al. 2020).

To reach the objective, a special sub-set of the dynamic panel data technique is applied to the 19 Eurozone countries from 2004 to 2018. It is the pooled mean group estimator (PMG) allowing for measuring in a single equation both the long-run relationship and the short-run speed of adjustment among variables. This approach, using the error correction form, delivers results considered to be consistent even in the presence of different dynamics of each country, despite the reduced number of explanatory variables and in presence of cross-sectional dependence.

The novel contribution of this paper lies in the perspective adopted, which aims at investigating if the policy structure of the Eurozone, based on a centralized monetary policy and national fiscal policies, is able to grant support to the political project of building up a cohesive continent. Therefore, it is a macroeconomic perspective, taking into account the effects of policy choices on growth and citizens’ general living conditions. In addition, the estimates measure the effect of the monetary policy strategy as a whole, concentrating not only on interest rates, but using a variable capturing the broad effect on monetary conditions occurring in the market as an effect of ECB intervention. As far as we know, this is the only contribution adopting this perspective, namely, evaluating policy effectiveness in contributing to EU institutional consolidation processes. Furthermore, the co-integrating technique employed allows us to register a stable and persistent long-run dynamic between the variables and therefore suggest the path to follow to regain consensus for the European project.

Our results reveal that the easy monetary policy strategy is associated with a decline of trust in the European Union and therefore that it was insufficient to counteract its decline and bring it back to pre-crisis levels. However, the switch of the ECB policy strategy that occurred after 2012 seemed to have exerted positive effects, although not enough to tell us that the trust in EU is a steady belief of Eurozone citizens.

These results allow for reflection on the policy strategies the European Union is going to implement to counteract the negative shock deriving from the COVID-19 pandemic. Without any institutional change, the policy architecture of the Eurozone based on centralized monetary policy and a strict national budgetary discipline—since it is not enough to sustain growth—seems to predict an increase of nationalist sentiment and a possible disintegration of the Eurozone.

The paper is organized as follows: Sect. 2 reviews literature about trust in European Institutions and its macroeconomic determinants. Section 3 presents the theoretical hypothesis behind our empirical model. Section 4 contains the empirical estimates and is divided into three subsections. Section 4.1 describes methodology; Sect. 4.2 contains the main estimates implemented following a baseline model and adding a set of control variables able to capture other determinants of trust while Sect. 4.3 provides a robustness check designed to follow the prevailing literature. Finally, Sect. 5 draws conclusions and offers policy implications for the management of economic policy in the Eurozone.

## The macroeconomic determinants of trust in institutions: the case of the Eurozone

The literature on trust in institutions is vast and goes beyond the scope of this paper to provide a comprehensive review. Since the 1970s, the “rationality hypothesis” and the centrality of “economic man” shifted the attention mainly toward the economic side of the analysis, which is at the centre stage of this investigation (for a complete review, see Nannestad and Paldman 1994). The results suggest that people: (a) are mainly “sociotropic”, that is, interested in the economic situation of the whole nation; (b) are retrospective with static expectations; and (c) assign the greatest importance to the unemployment rate (Veiga & Veiga, 2004). Here, we will focus on recent contributions dedicating specific attention to the case of the Eurozone.

The main focus of the analyses is the ECB because of its recognized role as a supranational institution. Empirical analysis starts in 1999 and applies panel data methodology. The first contribution concentrating on the relation between ECB performance and citizen trust is that of Fisher and Hahn (2008). Using Eurobarometer data from 1999 to 2004, they find—as is written in its institutional mandate—that the main issue defining trust in the ECB is the inflation rate. Surprisingly, the connection is positive—that is, the higher the inflation, the higher the trust (and vice versa). However, certain real variables, namely, gross domestic product (GDP) and unemployment, have to be taken into account. With the eruption of the financial crisis, the issue of trust and its links with the economic variables became increasingly important. Wälti (2012) empirically shows that the decline of trust in the ECB appears to be significantly evident in countries that have experienced increasing sovereign bond yields and financial turbulence. This leads to the apparently counterintuitive result that country-specific variables affect trust in a supranational institution. Through a micro-founded empirical model, and taking into account many factors influencing individual economic situations, Ehrmann et al. (2013) demonstrate that the decline in trust in the ECB is due to the combination of the following three effects: (1) the deterioration in economic conditions during the crisis; (2) the overall decline in public trust in the European project during the crisis; and (3) the fact that the ECB was associated with the troubles of the financial sector. However, they conclude that the evolution of the macro-economy is sufficient to explain the decline of trust and that there was not sufficient change in the regularities of the coefficient between normal and crisis times. Berlemann (2013) finds that the recent decline of trust in the ECB is attributable to financial and sovereign debt crises, even controlling for national macro-economic factors. Focusing on the institutional commitments of the ECB, Kaltenthaler et al. (2010) conclude that citizens’ lack of trust in the ECB is due to: (1) the deterioration of the economic situation; (2) the decline in belief in the European project; and (3) the association of the ECB with troubles in the financial sector (Kaltenthaler et al. 2010, p.10). The first two factors are also relevant to non-crisis times.

Studies with a wider institutional focus include those of Roth (2009) and Roth et al. (2011), which analyse the determinants of trust for the ECB, the EC and the European Parliament (EP). They consider in their estimates a set of macroeconomic variables. They conclude that unemployment and growth affect citizens’ trust, whereas debt and inflation do not have any effect during periods of economic distress (Hobolt, 2012). In subsequent studies, Roth et al. (2014), through a panel data analysis on 12 Eurozone countries, detect a negative and significant relationship between unemployment and trust in the ECB in times of crisis. They argue that the loss in trust is strongly driven by the significant increase in unemployment rates in four of the five peripheral countries: Greece, Ireland, Portugal and Spain.

Consistent with these results are those reached by Bonasia and Canale (2018), who find that trust in the three main European institutions (ECB, EC and EP) are, together with inflation and unemployment, strongly driven by fiscal measures implemented to comply with supranational rules (Canale & Liotti, 2018). In addition, Darkos et al. (2018) document a substantial negative impact on trust in ECB for countries experiencing a downgrade of their credit rating and participating in economic adjustment programmes (Muñoz et al 2011).

Focusing in particular on the effect on trust in the ECB of the interest rate setting policy in a sample ranging from 1999 to 2012, Albinowski et al. (2014) find a positive correlation between the two variables. The results are interpreted as a confirmation of the negative future prospects of the economy: ever-decreasing interest rates are the signal that the economy is further declining and therefore exerting negative pressure on trust in the ECB. However, in our opinion, this result can be interpreted also as citizens’ awareness of the inefficacy of monetary policy during declining macroeconomic conditions in the presence of fiscal retrenchments implemented to comply with rules.

One vein of research has identified factors affecting trust in the ECB among institutional issues. Horvath and Katuscakova (2016) examine whether trust in the ECB depends on transparency of its policy action. They find, through probit regressions with sample selections, that transparency exerts a non-linear effect on trust, since it increases trust, but only up to a certain point, above which transparency harms trust. This result is robust when controlling for a number of macroeconomic conditions, financial stability transparency measures and economic and socio-demographic characteristics of respondents. Farvaque et al. (2017) focus on the impact of socio-demographic factors on trust in the ECB, and find that people with higher education levels, people with centre-to right-wing political orientations and people with optimistic expectations on economic situations have higher trust in the ECB.

An interesting result is present in the work of Bergbauer et al. (2020), whose intent is to explain the opposite dynamics of trust in the common currency—the Euro—and trust in the ECB after the crisis. They find that the increasing support of the euro is predominantly value-based, while the decline in the trust in the ECB depends on its poor performance in affecting general macroeconomic conditions.

Armigeon et al. (2016) provide a general reflection affirming that the lack of support for both national and supranational governments in the Eurozone is weakening democracy. The main cause of the decline of trust resides in internal devaluation policies of the preceding years and in the impossibility of choosing between alternatives. Therefore the European Union project due to the economic crisis seem facing “existential” challenges (Hobolt & de Vries, 2016; Tosum et al. 2014) that need to be reconciled with national interests (Baldwin & Wyplosz, 2019; Frieden & Walter, 2017).

A recent updated research is the one contained in Roth et al. (2019). The authors enlarge the sample including all the countries that progressively joined the monetary union and, using panel data techniques, they conclude that the recent recovery in trust in European Union is mainly due to the reduction in unemployment.

Starting from these contributions, our aim is to verify the role played by the ECB in consolidating the process of integration of the European Union. The main rationale is that the action of monetary policy affects general macroeconomic conditions and therefore that citizen trust in the EU is highly affected by the efficacy of the measures implemented. In particular, beside unemployment and inflation, they affect, for example, fiscal stance, therefore defining the perimeter inside which, national fiscal policies can operate. Hence, we use broad ECB instruments to add additional elements to explain the dynamics of support in the EU’s institutional consolidation process.

## Monetary policy strategy in the Eurozone

The European policy model assigned the ECB the objective of price stability, as it is said to be the necessary condition for long-run convergence toward the natural unemployment rate. The achievement of the goal of stable inflation would have allowed perfect information, efficient market functioning and the achievement of full employment. With the objective of preserving the stability of the common currency, the ECB manages monetary policy for all the Eurozone member states. In its institutional mandate, the principle of “one size fits all” has been applied in the strong belief that financial integration assures real convergence. The ECB—the sole common economic policy institution—has in its hands, via monetary and interest rates tools, the responsibility to respect the inflation target and to ensure recovery of the Eurozone as a whole.

Starting from the financial crisis, the ECB implemented a number of measures to affect money market interest rates to encourage present consumption and investment against future spending. The lowering of the interest rates was achieved through changes in policy rates, through long-term refinancing operations and through security market programmes. In particular, to fulfil its missions as a liquidity provider, the ECB abandoned its bid-related main refinancing operations (MRO) in 2008 and instead adopted a fixed rate full allotment (FRFA) policy. To facilitate the access of commercial banks to funding, the ECB launched three programmes of covered bond purchases (CBPP 1, 2 and 3). To secure and stabilise the liabilities of commercial banks, the ECB extended the maturity of its long-term refinancing operations (LTRO) and then tendered very long-term refinancing operations (VLTRO) and targeted long-term refinancing operations (TLTRO 1, 2 and 3).

This manoeuvre should have closed the output gap, increased inflation and contrasted the increase in the real cost of funding in respect to the real returns of the market. However, these measures had the result of affecting the cost, rather than the amount of liquidity. Although targeting a low inflation rate has been successful at anchoring inflation expectations during the Great Moderation, it appeared to be insufficient in a very low real interest rate environment (Deutsche Bundesbank, 2017). As a matter of fact, there is not enough room for central banks to reduce policy rates in response to an economic downturn and preserve a homogeneous monetary policy transmission mechanism across countries (European Parliament, 2019). With the rise of the European government bond crisis, the increase in sovereign yields hampered the transmission of the common monetary policy to the real economy. In this context, the ECB launched a series of assets purchases: the securities market programme (SMP) and the asset-backed securities (European Parliament, 2019).

In 2012, the new Governor Mario Draghi announced that he would have saved the Euro “whatever it takes”, and the monetary policy strategy was enriched by further unconventional instruments trying to directly affect the amount of liquidity in circulation. Together with very low policy rates, the ECB started to expand the balance sheet as an additional policy instrument to inject liquidity into the market. The asset purchase programme was extended to the public sector without additional sterilization interventions and with the intention to keep them in the balance sheet until maturity. The long-term refinancing operations were prolonged, and a two-tier system for bank reserves remunerations was defined. At present, the deposit facility is at − 0.50%, the main refinancing operation rate is at zero, and the marginal lending facility is at 0.25%. Through this variety of instruments, the monetary policy strategy was enriched by an instrument of “forward guidance”, explicitly signalling to the market that interest rates will remain low for a prolonged period of time. Furthermore, the direct purchase of public bonds lowered long-term government bond yields of peripheral countries, reducing the spread and supporting the fiscal space of countries in difficulty.

Based on the evolution of the monetary policy strategy, we investigate the role of ECB in the decline of support of the EU using a variable capturing the monetary policy strategy as a whole, namely, the yield curve. The yield curve provides valuable information on the impact of monetary policy on the real economy measures and allows for investigation of how policy rate changes and unconventional measures shape the credit market and affect the real economy (Deutsche Bundesbank, 2017).

The yield curve is a key determinant of the financing conditions of the economy and a central element in the transmission of monetary policy. It is affected contemporaneously by policy interest rates and public and private bond purchase programmes and therefore provides information about the true safe rates present in the market and their potential influence on the real economy (Lane, 2019).

**Fig. 2:**
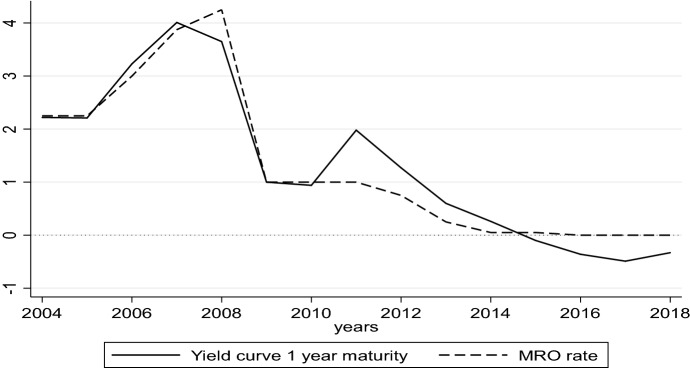
One-year maturity yields on safe government bonds and interest rates on main refinancing operations. *Source*: Own calculation on ECB data

Figure 2 provides a comparison between the rate of the main refinancing operation calculated as a yearly average and the interest rates on government bonds rated AAA+ extracted for each year from the yield curve with one-year maturity. It helps to understand the differences in capturing the monetary policy strategy of the two variables (see also Deutsche Bundesbank, 2017, p. 17).

As is shown, from the beginning of our sample and until the financial crisis, the two rates follow almost the same path. The sovereign bond crisis in 2010 caused an increase in sovereign bond yields that the monetary policy strategy did not get under control, as the progressive lowering in interest rates was not enough to drain liquidity into the market. However, in subsequent years, the difference between the two rates started to decline, reaching a very moderate distance in the year 2013. From 2015 to now, interest rates on government bonds became negative, with the line describing its path standing below the zero line of the MRO policy rate. These dynamics show a marked change in monetary policy strategy that became expansive from 2013. Our hypothesis is that monetary policy became more effective, causing a wider fiscal space and a massive injection of liquidity that started to reach households and firms.

## Empirical analysis

**Fig. 3:**
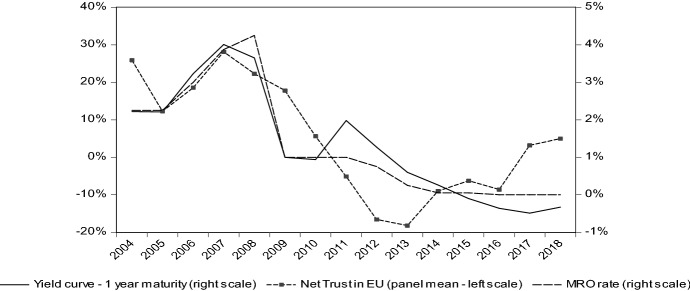
Net trust in EU project and monetary policy strategy. *Source*: Own calculation on Eurobarometer and ECB data

This paper focuses on 19 European countries (Austria, Belgium, Cyprus, Estonia, Finland, France, Germany, Greece, Ireland, Italy, Latvia, Lithuania, Luxembourg, Malta, Netherlands, Portugal, Slovenia, Slovakia and Spain), adopting common currency and therefore a common monetary policy. The time span ranges from 2004 to 2018. They represent a homogeneous sample to evaluate the effect on trust in the EU regarding monetary policy choices. Since data about trust in European Union project are available for all countries considered from 2004, backdating the sample to the birth of the monetary union would mean to consider an unbalanced panel, assigning a heavier weight so some countries in respect to others. Trust in the EU, collected from the standard Eurobarometer survey, is considered as the dependent variable. The Eurobarometer is a survey established in 1973, which has been progressively refined in the course of the years. Each survey consists of approximately 1000 face-to-face interviews per Member State, and reports are published twice yearly. It is structured around a wide range of questions. The question this paper is concerned about is: “Do you tend to trust in European Union?”^2^ The respondents have three options: (1) “tend to trust”; (2) “tend not to trust”; and (3) “don’t know”. Our measure of trust has been calculated as the difference between the percentage of the total population that tends “to trust” and the percentage of the total population that tends “not to trust” (Walti 2012).^3^ As the standard Eurobarometer is a biannual survey, the simple average of the two observations available for each year has been calculated. The main explanatory variable is the interest rate extracted from the one-year maturity yield curve of government bonds rated AAA+. As described above, it is a variable capturing the monetary policy strategy over the policy rates, as it indirectly better captures the macroeconomic effects of ECB interventions. Figure 3 presents a first insight about the relation between the panel mean indicator of trust and the monetary policy strategy. It obtained joining Figs. 1 and 2 and therefore depicting the supposed connection in a single picture. In appendix, descriptive statistics of the main variables enrich the description.

Following this first insight and to evaluate the change in the monetary policy strategy from 2013 to the present, an interaction dummy has been introduced, obtained by multiplying the value of one by the yields for the period ranging from 2013 to 2018, and zero otherwise. It allows for separation of the effect of conventional monetary policies implemented through interest rate setting from those derived from massive injections of liquidity as a consequence of open market operations and ECB balance sheet expansion. It allows, therefore, for capture of the specific effect of the “unconventional” monetary policy (adopted by the former ECB chief Mario Draghi) on trust in EU. Data are collected from the ECB database (https://sdw.ecb.europa.eu/browse.do?node=9691126).

Furthermore, a set of control variables derived from the literature has been introduced: (1) GDP growth; (2) inflation rate (Paldam, 2004; Nannestad and Paldman 1994 and many others); (3) a dummy variable capturing the presence of a debt above the thresholds allowed, as a signal of restrictive fiscal policies and financial instability—it assumes the value of 1 if the ratio is higher than 60% and zero otherwise; and (4) the labour share of GDP. This indicates the percentage of GDP going to employees: it can be considered a measure of income distribution that is unaffected by country specific measures against inequality and a degree of workers’ involvement in GDP. Data about control variables are retrieved from Eurostat database (https://ec.europa.eu/eurostat/data/database).

### Methodology

The panel dynamic empirical technique applied is the pooled mean group estimator (PMG) and relies on the existence of a long-run relationship among the variables and on the model convergence toward an equilibrium value. This is due to the error correction form of the model (ECM) that estimates separately the coefficients of the variables in a dynamic form with lagged values (long run) and the coefficient of the dynamics of the adjustment process (short run). Furthermore, the ECM provides the speed of adjustment, which is supposed to assume a value that is negative and lower than one, proving that there is a short-run dynamic of adjustment toward a long-run equilibrium value. The precondition to apply this methodology is that the main variables of the model need to be non-stationary in their level, integrated of the same order and co-integrated. Therefore, it represents a required choice to in presence of non-stationary variables, that when included in other panel estimators—such as the panel ordinary last square (OLS), the generalized method of moments (GMM) or the feasible generalized last square (FGLS)—provide misleading results. In respect to other panel dynamic techniques (PDOLS), the PMG follows the error correction (EC) form and supports the existence of a stable connection trough time among the variables, in presence of correlation among the explanatory variables, endogeneity issues and heterogeneity across panel members. In addition, it accounts for cross sectional dependence, feature to be considered of the utmost importance for spatial and temporal strictly interconnected entities.

Following the PMG estimator, short-run coefficients are allowed to vary across groups, while long-run parameters are constrained to be equal (Blackburne & Frank, 2007; Pesaran & Smith, 1995; Pesaran et al. 1999). Since this implies co-integration, it allows for the individuation of the eventual presence of a stable relationship, even in the presence of a reduced number of explanatory variables. In respect to other panel dynamic techniques, the PMG provides therefore long-run consistent results in presence of a number of inefficiencies attributable to other panel estimators. The features of PMG can be considered to be consistent for dynamically estimate the supposed connection for the sample of 19 Eurozone countries, in which the presence of cross-sectional dependence and different adjustment dynamics in each country could lead to misleading results. Furthermore, the empirical model accounts for the implicit presence of dummy variables, as the individual-specific regressors are filtered by means of cross-section averages. In this way, the differential effects of unobserved common factors (such as the 2007 financial crisis) are eliminated (Pesaran, 2007).

The equations to be estimated assume the long- and the short-run forms. The long-run equation follows the ADRL process using current and past values of the explanatory variables and is described by:

1 $$ \begin{aligned} NT\_EU_{i,t} & = \alpha_{i} \, + \, \lambda_{i,j} NT\_EU_{i,t - 1} \, + { (}\beta_{i,0} + \beta *_{i,0} )YC_{i,t} \\ & \quad + {(}\beta_{i,1,} + \beta *_{i,1} )YC_{i,t - 1} \, + \, \gamma_{i,0} X_{i,t} \, + \, \gamma_{i,1} X_{i,t - 1} { + }\varepsilon_{i,t} . \\ \end{aligned} $$

This is the long-run specification equation, where NET_EU is net trust in EU, while YC is the annual government bond yield extracted from the yield curve of bond rated AAA+, X is the set of control variables inserted one by one, and *i* represents the country. In Eq. (1), β_0_ and β_1_ are the coefficients of the main independent variable for the whole period considered, while β_0_*and β_1_* are the dummies on the coefficient, such that the values of β_i_* assume the value of zero before the year 2013 and represent the estimated coefficients for the last five years of our sample. Therefore, the effect of the switch in monetary policy strategy is measured by the parameters (β + β*). This allows the sample not to be split into a pre- and post-crisis period, overcoming the well-known limitations of having to compare results from two separate samples, and gaining in terms of robustness of the parameter estimates. According to the ECM form, the residuals coming out of the long-run equation are then used to verify the long-run convergence toward the equilibrium value or to verify, as it is called, the speed of adjustment. Therefore, in the short-run, changes in the dependent variables should depend on changes in the independent variables, plus an error term measuring if they converge. Thus, the error correction equation describing the short-run speed of adjustment is:

2 $$ \Delta NT_{i,t} \, = \, \phi_{i} (NT_{i,t} \, - \, \vartheta_{i} \, - \, \vartheta_{1,i} YC_{i,t} - \vartheta_{2,i} X_{i,t} ) - \beta_{i,1} \Delta YC_{i,t} \, - \gamma_{i,1} \Delta X_{i,t} + \, \mu_{i,t} , $$

where, with simple transformations, it is easy to verify that:

$\vartheta_{i} \, = \, \frac{{\alpha_{i} }}{{1 - \lambda_{i} }}$,$\vartheta_{1,i} \, = \, \frac{{(\beta_{i,0} + \beta *_{i,0} ) + (\beta_{i,1} + \beta *_{i,1} )}}{{1 - \lambda_{i} }}$, $\vartheta_{2,i} = \frac{{\gamma_{i,0} + \gamma_{i,1} }}{{1 - \lambda_{i} }}$ are the long-run coefficients calculated as a weighted average of the coefficient of the independent variables.

The weight is given by the coefficient of the dynamic dependent variable, and $\phi_{i} = - (1 - \lambda_{i} )$ is the error correction speed of adjustment. In the estimates, it has to be significant and $- 1 < \phi_{i} { < 0}$.

The parameters $\vartheta$ for the long-run, $\beta$ and $\beta *$ for the short-run and $\phi_{i}$ for the speed of adjustment are the relevant parameters to be estimated in the model. They are estimated with and without the introduction of control variables to support the hypothesis of a direct link between net trust in the EU and monetary policy strategy in the long-run. The adoption of the dynamic panel empirical model described above depends on its technical properties or on its ability to provide reliable estimates for non-stationary variables and heterogeneous panels, as is the case of our sample, even in the presence of a reduced number of explanatory variables and endogeneity issues. Furthermore, the long-run approach provides support to the hypothesis about the existence of a stable relationship between net trust and monetary policy and causes us to reflect on the efficacy of the policy instruments in the Eurozone.

### Results

The first step of the empirical analysis investigates the properties of our panel data to choose the appropriate methodology for stationarity and cointegration tests. In presence of cross-sectional dependence, panel second-generation tests should be applied. This circumstance is related only to the main dependent variable NT, as it is the sole variable to have panel features. YC, on the contrary, as it is the proxy of the common monetary policy strategy, is the same for all countries and therefore should be treated as a simple time series. The Pesaran (2004) CD test for NT_EU is 31.674***, therefore rejecting the null hypothesis of no-cross sectional dependence.

The cross-sectional dependence in series suggests using the so-called “second generation” test to investigate the presence of a unit root in NT_EU or the CADF panel unit root test (Pesaran, 2007). If the null hypothesis is rejected, the series is stationary. Stationarity for YC are tested using time series methodology, and therefore the DF-GLS test is applied (also known as the ERS test from Elliot et al. 1996). Panel A in Table 1 presents the results.

**Table 1:** Unit root and cointegration tests for NT_EU and YC

Panel A	Unit root tests	
	CADF Test	
Variable	Individual effect	Individual effect and trend
NT_EU	− 0.214	− 0.001
ΔNT_EU	− 6.774***	− 5.630***
	DF-GLS test	
YC	− 0.956	− 2.798
ΔYC	− 4.476***	− 4.534***

Panel B	Westerlund cointegration test between NT_EU and YC		
Statistic	Value	Z value	p value
G_t_	2.399	2.971	0.002
G_a_	− 11.972	3.837	0.000
P_t_	− 9.857	3.385	0.000
P_a_	− 13.123	8.328	0.000

***, **, and *Reject the null at 1%, 5% and 10% respectively. NT_EU is net trust in European Union, YC is government bond returns extracted from 1 year maturity yield curve

The tests are performed both for individual effects and for individual effects and trend: the null hypothesis of no stationarity is accepted for the variables in their level, while rejected when considering NT_EU and YC at first differences. Therefore, it is possible to support the conclusion that the net trust in the EU and yields on government bonds are integrated of order one.

To verify the presence of a long-run relation between net trust in the EU and YC, the Westerlund (2007) “second generation” cointegration test accounting for cross-sectional dependence is performed. Table 1, Panel B reports the results. The G_t_ and G_a_ test statistics checks the null hypothesis of no cointegration for each cross-sectional unit, while the P_t_ and P_a_ test statistics pool information over all the cross-sectional units and follow the same null of the previous two tests. The null hypothesis of no cointegration is rejected for all of the four tests.

The presence of cointegration gives strong support to the results of the estimation of the dynamic panel model presented in Table 2. The estimation procedure is articulated in two phases: a first phase in which the pure relation between the dependent variable (NT_EU) is estimated to be dependent on the explanatory variable of the model (YC); and a second phase in which the estimations are replicated, adding the control variables one at a time to avoid misleading results due to the reduced observations number. These control variables are: a dummy on yield curve for the period 2013–2018 which is maintained for the all subsequent models (D_YC); the growth rate (GR); inflation (INF); labour share on GDP (LS); and a dummy variable connected to the debt/GDP ratio assuming the value of one when debt is above the threshold of 60% and therefore signalling restrictive fiscal measures or financial instability, and zero otherwise (D_DEBT).

**Table 2:** Trust in European Union and ECB monetary policy: dynamic panel data analysis with PMG estimator

Independent variables	(I)	(II)	(III)	(IV)	(V)	(VI)
Long run						
YC	5.240*** (1.079)	6.990*** (0.915)	10.478*** (0.775)	11.316*** (1.144)	10.685*** (0.652)	10.101*** (0.741)
D_YC		− 23.145*** (5.532)	− 15.672*** (4.642)	− 23.768*** (5.971)	− 13.415*** (4.065)	− 13.501*** (4.319)
GR			8.211*** (0.954)	9.383*** (1.187)	7.810*** (0.934)	6.719*** (0.741)
INF				− 2.654*** (2.255)		
LS					1.575** (0.719)	
D_Debt						− 11.957*** (3.845)
Short run						
ϕ_i_	− 0.327*** (0.044)	− 0.372*** (0.055)	− 0.346*** (0.058)	− 0.308*** (0.046)	− 0.414*** (0.063)	− 0.376*** (0.063)
ΔYC	0.545 (0.708)	− 0.377 (0.761)	− 6.058*** (1.163)	3.861** (1.275)	− 8.575*** (1.504)	− 5.627*** 1.117
ΔD_YC		− 4.263* (2.529)	0.582 (2.533)	1.332 (2.818)	− 1.373 (3.229)	0.205 (2.583)
ΔGR			− 0.189 0.187)	− 0.237 (0.194)	− 0.071 (0.245)	− 0.173 (0.163)
ΔINF				− 1.743*** (0.506)		
ΔLS					0.317 (0.783)	
ΔD_Debt						− 0.543 (0.834)
Groups	19	19	19	19	19	19
Observations	266	266	266	266	266	266

Dependent variable: Net trust in European Union (NT_EU)

***, **, and *Reject the null at 1%, 5% and 10% respectively. YC is government bond returns extracted from 1 year maturity yield curve; D_YC is the interaction dummy for the period 2013–2018 to account for the switch in monetary policy; GR is the GDP rate of growth; INF is the inflation rate; LS is the labour share of GDP; and D_Debt is the dummy variable assuming a value of 1 when debt/GDP ratio is above the threshold of 60%

The first thing to be noted is the goodness of the methodology adopted, since in all the models considered, $- 1 < \phi_{i} < 0$ and highly significant.

Column 1 shows the result for the baseline model representing our benchmark (model I). In the long run, monetary policy is positively connected with trust in the EU (5.260***), showing that a decrease in interest rates causes a decline of trust. The decrease in policy rates is interpreted as a confirmation of declining expectations (Albinowski et al. 2014), and as the occurrence of conditions of a “liquidity trap”—namely, the inability of the sole monetary policy to stimulate the economy during declining macroeconomic conditions (Schmitt-Grohé & Uribe, 2012). In the short run, the effect of the yield curve on trust is positive but not statistically significant. This outcome does not void the long run result as it is deeply affected by the different processes of adjustment occurring in each individual panel member.

In models II, III, IV and VI control variables are introduced into the baseline model. The results obtained in model I for the main explanatory variable are replicated in subsequent models too (6.990*** for model II, 10.478*** for model III, 11.316*** for model IV, 10.685*** for model V, and 10.101*** for model VI. It is noteworthy that in the last three models, the coefficients are very similar. An additional very important result is obtained when the interaction dummy on 1-year maturity government yields is added as a control variable. It is able to capture the differences in the policy strategy after 2013 without splitting the sample and losing the informative power assured by the number of observations. Although results about the coefficient of YC are not affected significantly, a change in the sign of D_YC is reported. In fact, the specific coefficient of the interaction dummy on yields is negative, which, added to the positive value of the main explanatory variable, still gives us a negative result (6.990*** − 23.145*** = − 16.155 for model II, 10.478*** − 15.672*** = − 5.194 for model III, 11.316*** − 23.768*** = − 12,452 for model IV, 10.685*** − 13.415*** = − 2.73 for model V, 10.101*** − 13.501*** = − 3.04 for model VI). This is a signal of an opposite effect on trust of the monetary policy strategy for the last five years of our sample. The change in strategy was perceived as more effective when implemented not only through changes in policy rates, but also through a variety of liquidity instruments enhancing credit conditions and improving governments’ fiscal space. This result shows that the adoption of an “unconventional” monetary policy has been able to invert the decline of trust towards the EU project.

In model III, economic growth is added as a control variable. As the literature predicts, the sign of the long-run effect has a concordant sign (8.211***), confirming growth as one of the most important variables to take into account to evaluate trust dynamics (similar results are present also in model IV, 9.383*** model V, 7.810*** and model VI, 6.719***). Model IV includes inflation: as expected, the long-run sign is negative and equal to − 2.654**. Despite in a reduced way in respect to growth, the value of the long-run panel coefficient of price growth tells us that inflation is a cause of concern for citizens. Model V adds the labour share. The coefficients of the other main variables are not affected significantly, and the specific coefficient is positive and significant (1.575**), supporting the conclusion that workers assign to the EU the responsibility of a declining labour share, and that therefore, when workers’ participation in GDP increases, trust increases, too (and vice versa). This result seems to support the point of view that a more equal share of output supports the EU’s institutional consolidation process. Finally, in model VI, the dummy variable signalling a debt above the threshold is added. Its long-run value is − 11.957***, showing that when debt is above the 60% threshold, trust decreases. This negative relationship can be due to both a negative evaluation of EU thresholds and a perception that fiscal retrenchments that are necessary to reduce public debt are causing a worsening of general living conditions.

When examining results of the short-run process of adjustment, it is noteworthy that the sole main explanatory variable—that is, yield—is significant. The goodness of the model is not compromised, as the value of $\phi_{i}$ is always below zero and above minus one, and therefore the long-run relationship between the variables in their levels is valid. On the contrary, this can be interpreted as proof that the process of adjustment in the short run follows different dynamics in each panel member. Furthermore, in the short run, the sign of the coefficient of the yield has an opposite sign in respect to the long-run estimations. This helps to support the conclusion that when considering changes, trust in the EU was supported by the expansionary monetary policy strategy, although it was not considered sufficient to support the project in the long run.

### Robustness check

In order to account for the literature prevailing results about the main determinant of trust, we investigated if the relation between the monetary policy stance and support to the European Union is still valid in presence the contemporaneous consideration of the most relevant variables detecting general macroeconomic performance of each single country (Roth et al. 2019 for EU). As robustness check, we include therefore together with growth, unemployment and inflation in the main estimates. The long-run estimated equation is the same of Eq. (1) transformed according to the joined inclusion of these new control variables.

Table 3 shows that the general validity of the proposed model does not change as the coefficient of YC is positive (11.284***) and the one of D_YC is negative (− 15.6***) and high in absolute value. In accordance with the authoritative literature on the subject, growth and unemployment are key factors in defining the citizens’ support to the European Union both in the long and short run. In particular, a raise of one percent point in the unemployment rate reduces the trust in European Union project by − 0.99*** in the long run and − 3.159** in the short run. Consistently, GDP growth increases trust in the long run (3.259***) while nothing can be said for the short run. When observing inflation rates results are mixed as coefficients in the long run are positive (1.478***) while in the short-run negative (− 2.268***). This last result can be interpreted through citizens’ long run perception of negative prospects of the economy. It is worth noting that the EC term is ϕ_i_  = − 0.402*** negative and greater than -1, therefore confirming the validity of the chosen empirical model.

**Table 3:** Trust in European Union, ECB monetary policy strategy and macroeconomic performance

Independent variables	
Long run	
YC	11.284*** (0.520)
D_YC	− 15.600*** (2.467)
GR	3.259*** (0.535)
UN	− 0.993*** (0.085)
INF	1.478*** (0.301)
Short run	
φ_i_	− 0.402*** (0.079)
ΔYC	1.557** (0.887)
ΔD_YC	− 6.564*** (1.247)
ΔGR	− 0.014 (0.404)
ΔUN	− 3.159** (1.280)
ΔINF	− 2.268*** (0.545)
Constant	− 4.259*** (1.428)
Groups	19
Observations	266

Dynamic panel data analysis with PMG estimator. Dependent variable: Net trust in European Union (NT_EU)

***, **, and *Reject the null at 1%, 5% and 10% respectively. YC is government bond returns extracted from 1 year maturity yield curve; D_YC is the interaction dummy for the period 2013–2018 to account for the switch in monetary policy; GR is the rate of growth, UN is unemployment and INF is the inflation rate

From these results, it can be derived that, besides monetary policy strategy, variables capturing social and economic single countries conditions are of the utmost importance in defining trust in the European project.

## Conclusions

Trust is a very important feature of democratic institutions, as it measures the perception of how such institutions serve the public interest. The institutional structure of the Eurozone offers a very particular field of investigation, allowing the degree of integration of national economies into a supranational cohesive framework to be evaluated.

Starting from the observation of a general decline of trust in Eurozone countries, the aim of this paper was to evaluate the ability of the monetary policy strategy in supporting trust in the EU and therefore in a solid and cohesive supranational project. Our analysis moves from the premise that the ECB is the sole supranational policy institution, with the task of serving multiple kinds of national interests. National fiscal policies—to be managed in a general criterion of spending constraint—are associated with this supranational strategy. In this context, conflicts may arise, and the ECB can fail to reach the objective of preserving the existence of the monetary union. Results support the conclusion that when monetary policy follows the simple strategy of interest rate setting, it is unable to support trust in the EU, while when it intervenes with a variety of instruments to support the injection of liquidity and national fiscal policies, its efficacy appears to be greater as an inversion of the decline of trust is registered. This outcome is robust even in the presence of a variety of control variables accounting for specific national situations of growth, unemployment, inflation, compliance to rules and distribution of income.

During declining macroeconomic conditions and in the presence of a liquidity trap, the sole interest rate setting strategy is not able to stimulate aggregate demand. When the instruments become more complex and capable of intervening on other components of aggregate demand, the accumulation of reserve liquidity reduces, supporting national fiscal space, private investments and consumption. In brief, the ECB is able to support the EU project when it is able to improve national macroeconomic performance with an effective, although indirect, contribution to aggregate demand.

However, the inversion of the decline of net trust in the EU registered in recent years is slight, as on average, it is a little above the value of zero and far from the initial positive value. Therefore, further instruments would be required to build up a solid and viable European Union, which is capable of serving the interests of its citizens.

The time span under investigation in this paper covers a period during which the systemic shock coming from the crisis transformed into an asymmetrical shock, causing centripetal forces in the European Union to arise. The monetary policy strategy introduced after 2012 gave a fundamental contribution to citizen confidence toward the EU project. The instruments were able to smooth differences across countries and to close the spread between different public bonds. The results obtained can be used, therefore, to evaluate the possible scenarios and the appropriate policy response to the systemic shock caused by the Covid-19 pandemic. It originated as a systemic shock, but threatens to turn into an asymmetric shock, because of the single countries’ degrees of resilience. The experience of the post-crisis period suggests searching for a common policy response to avoid a permanent loss of trust in the EU project.

However if the ECB, under Lagarde’s presidency, is implementing a set of extraordinary measures in line with the Draghi mandate—among which the Pandemic Emergency Purchase Programme (PEPP) with €1350 billion is the most important measure—little can be said about a common response through coordinated fiscal policy action. The most promising instrument is the “recovery fund”, through which the EC is planning to sustain countries hit by the Covid-19 shock. The results presented in this paper allow us to draw a lesson from the past, informing us that extraordinary monetary policy measures help to sustain the European institutional consolidation process. However, they can be not enough. Therefore, whatever the result of the bargaining, national and supranational policy-makers and institutions should be aware that the true object of discussion is the survival of the EU political project.

## Appendix

See Table

**Table 4:** Summary statistics for Net trust in EU, MRO rate and yield curve

Variables	Obs	Mean	Std. dev	Min	Max
Net trust in EU	285	4.99	23.70	− 61.59	53.29
MRO	285	1.31	1.41	0	4.25
Yield curve	285	1.33	1.44	− 0.49	4.01

^4^.

## Footnotes

1 The standard Eurobarometer was established in 1973. Each survey consists in approximately 1000 face-to-face interviews per Member State (except Germany: 1500, Luxembourg: 500, United Kingdom 1300 including 300 in Northern Ireland) and reports are published twice yearly. The entire dataset containing results about the whole set of questions is available at https://ec.europa.eu/commfrontoffice/publicopinion/index.cfm/Chart/index.

2 To be precise, the question is formulated in the following way: “For each of the following media and institutions, please tell me if you tend to trust it or tend not to trust it”. Of course, we consider the EU institution.

3 In the literature, a measure of net trust as a percentage of total population that includes those who don’t know’ is also used (Roth 2009; Roth et al. 2011 and 2014). However, we chose to exclude this considering that they do not have enough instruments to express a judgment.
